# A PVDF Receiver for Acoustic Monitoring of Microbubble-Mediated Ultrasound Brain Therapy

**DOI:** 10.3390/s23031369

**Published:** 2023-01-26

**Authors:** Yi Lin, Meaghan A. O’Reilly, Kullervo Hynynen

**Affiliations:** 1 Department of Medical Biophysics, University of Toronto, Toronto, ON M5G 1L7, Canada; 2 Physical Sciences Platform, Sunnybrook Research Institute, Toronto, ON M4N 3M5, Canada; 3 Institute of Biomedical Engineering, University of Toronto, Toronto, ON M5S 3G9, Canada

**Keywords:** hydrophone, blood–brain barrier, focused ultrasound (FUS), micromachining, transcranial therapy

## Abstract

The real-time monitoring of spectral characteristics of microbubble (MB) acoustic emissions permits the prediction of increases in blood–brain barrier (BBB) permeability and of tissue damage in MB-mediated focused ultrasound (FUS) brain therapy. Single-element passive cavitation detectors provide limited spatial information regarding MB activity, greatly affecting the performance of acoustic control. However, an array of receivers can be used to spatially map cavitation events and thus improve treatment control. The spectral content of the acoustic emissions provides additional information that can be correlated with the bio-effects, and wideband receivers can thus provide the most complete spectral information. Here, we develop a miniature polyvinylidene fluoride (PVDF thickness = 110 μm, active area = 1.2 mm^2^) broadband receiver for the acoustic monitoring of MBs. The receiver has superior sensitivity (2.36–3.87 V/MPa) to those of a commercial fibre-optic hydrophone in the low megahertz frequency range (0.51–5.4 MHz). The receiver also has a wide −6 dB acceptance angle (54 degrees at 1.1 MHz and 13 degrees at 5.4 MHz) and the ability to detect subharmonic and higher harmonic MB emissions in phantoms. The overall acoustic performance of this low-cost receiver indicates its suitability for the eventual use within an array for MB monitoring and mapping in preclinical studies.

## 1. Introduction

Microbubble-mediated focused ultrasound (FUS) has been exploited as a method for brain therapy, including targeted delivery of various agents [1,2,3,4,5,6,7,8], and has now reached clinical trials for a number of indications [9,10,11,12,13]. Successful agent delivery is believed to be caused by interactions between the acoustically stimulated microbubbles (MBs) and the walls of the blood vessels, temporally increasing the blood–brain barrier (BBB) permeability, termed BBB modulation (BBBM) [14]. Currently, treatment assessment involves using contrast-enhanced magnetic resonance imaging (MRI) to evaluate the change in BBB permeability, as well as susceptibility weighted or T2* weighted MRI, to detect potential hemorrhage. However, MRI is usually performed after sonication and cannot monitor the treatment in real time. MRI may not be capable of capturing spontaneous vascular damage as it may take tens of seconds to reveal [15]. As over exposure is often correlated with undesired tissue damage [16] and under exposure with ineffective therapy, a real-time control method is critical in ensuring treatment safety and efficacy.

One method of directly controlling FUS-induced BBBM is through monitoring MB emissions. MBs are preformed lipid- or albumin-encapsulated gas bubbles commonly used as a contrast agent for ultrasound imaging [17]. MB activity is pressure-dependent [18]. Upon the excitation of ultrasound waves at a particular frequency (f0) and an initial acoustic pressure [19], MBs will scatter ultrasound at the fundamental and harmonic frequencies ((*n* + 1) f0, *n* = 1, 2, 3…). With increasing pressure, an MB will expand and compress in the stable cavitation (SC) regime. If pressure passes a threshold, subharmonic ((1/(*n* + 1)) f0, *n* = 1, 2, 3…) and ultraharmonic (((2*n* + 1)/2) f0, *n* = 1, 2, 3…) emissions can be observed. With further increase in pressure amplitude, the MB enters the inertial cavitation (IC) regime. The oscillatory behavior becomes more violent until the MB collapses, resulting in broadband emissions. These spectral characteristics allow types of cavitation to be distinguished [20].

The changing spectral characteristics of the MB emissions with increasing pressure correlate well with FUS-induced BBBM bioeffects. The IC behaviour of the MBs has been shown to be associated with vascular damage, in addition to changes in BBB permeability. In particular, the presence of broadband emission, a signature of IC, has been correlated with erythrocytes extravasation [21] and vascular damage [22]. Thankfully, BBBM can also be induced without IC at a lower pressure level [23]. Harmonic emissions have been correlated to MR contrast enhancement, indicative of the successful and possibly proportional degree of BBBM in non-human primates [24]. Proportional relationships between harmonic emissions and safe BBBM duration, permeability, and likelihood have also been reported [25].

Broadband emission is a signature of IC and associated with unwanted bioeffects. Thus, safe BBBM requires remaining below the IC threshold [26,27]. Acoustic emission-based controllers can be broadly grouped into two types based on the non-inertial cavitation signatures that they analyze. The first type focuses on the detection of sub- and/or ultraharmonics as the appearance of these are threshold events and their onset occurs as the IC threshold is being approached [28]. O’Reilly et al. established the first BBBM control algorithm in 2012, where tissue damage was avoided by reducing pressure to a 50% level upon the detection of sub- or ultraharmonic emissions using a customized receiver [29]. Different integration methods for establishing a subharmonic threshold value were also investigated in pig through ex vivo human skull in preparation for clinical studies [30]. More recently, this algorithm has been extended to receiver arrays that can generate spatial maps of bubble activity [31]. Another subharmonic thresholding algorithm of BBBM control was suggested by Tsai et al., where the threshold detection serves as a surrogate to turn on/off FUS exposure as a means of control [32]. 

The strength of harmonic emissions has also been proposed as a measure to define safe treatment [23,24,26]. A closed-loop proportional-integral controller for safe BBBM was suggested by Sun et al. [15]. They first characterized harmonic and broadband emissions at different pressure amplitudes using a range of MB doses. Then, they selected the optimal MB dose that produced the most linear harmonic emissions to pressure response, and maximum harmonic emission amplitude without broadband emission to establish the largest control window. The harmonic emission threshold for safely inducing BBBM was then determined in a pilot experiment of a rat glioma model. 

The aforementioned controller algorithms either rely on the detection of sub- and/or ultraharmonics, or thresholding pressure using harmonic emissions. The bandwidths of the receiver used in those approaches were, for the most part, not sufficiently wide to detect multiple signatures. Therefore, combining the two approaches to calibrate and modulate BBBM pressure using multiple characteristic emissions remains challenging.

Another drawback of the single-receiver design is spatial specificity. Studies have been performed using a set of three to four hydrophones to localize cavitation activity in tissue [33] and through ex vivo human skull [34]. The minimal hydrophone approach was shown to be feasible in localizing cavitation cloud sources in 2D space with a few mm localization accuracy for sources 30 mm from the geometric center of the sensor network [34]. However, 3D spatial information is critical for verifying the cavitation event in space to ensure precise targeting and avoid tissue damage, especially in small animals where higher accuracy may be required. For systems operating at clinically relevant frequencies, commercial linear arrays and customized receiver arrays have been used to implement passive acoustic mapping (PAM) to obtain spatiotemporal information of MB activity using different beam-forming algorithms [35,36,37,38,39]. Commercial linear arrays with broad frequency bands are limited in that they can only generate 2D maps [40]. The achievement of 3D mapping has been brought about by using a clinical scale custom hemispherical array [41]; however, the receiver frequency bandwidth limits the achievable resolution when mapping MB clouds. Whereas these receiver systems satisfy the millimeter resolution requirement for clinical studies, pre-clinical studies require higher spatial resolution for precise anatomical targeting and MB monitoring to match small animal brain structures. 

To map MB activity for preclinical studies, a high frequency receiver array with small aperture broadband elements to reduce directivity is needed to provide high spatial resolution at all characteristic frequencies. Commercially available calibrated hydrophones, such as fibre-optic and needle hydrophones, are either expensive and/or bulky and thus do not satisfy the specific needs of monitoring ultrasound brain therapy. In this paper, we present a small aperture and a broadband receiver operating in the low megahertz (MHz) range with minimal fabrication expenses. Sensitivity, minimum detectable pressure, and directivity were characterized with free-field acoustic measurements. The acoustic characteristics were compared to those of commercial fibre-optic and needle hydrophones, and an in-house lead zirconate titanate (PZT) receiver previously used in vivo studies. Benchtop bubble experiments were conducted to examine the receiver’s ability to detect MB activity compared to that of the PZT receiver.

## 2. Materials and Methods

### 2.1. Receiver Assembly

A miniature receiver was fabricated using 110 μm polyvinylidene fluoride (PVDF) film (Measurement Specialties Inc., Hampton, VA, United States of America) with nickel-copper electrodes (700 A Cu, 100 A Ni), having an active area of 1.1 mm by 1.1 mm. PVDF film was selected for its well-known broadband characteristics over common lead zirconate titanate (PZT) material. The active layer was backed with 1.35 mm thick conductive silver epoxy (GPC-251LV, Creative Materials Inc., Tyngsboro, MA, United States of America) mixed with a 10% volume ratio of tungsten powder (Micron Metals Inc., Upper Saddle River, NJ, United States of America) to improve bandwidth and increase sensitivity in low MHz range [42]. Conductive silver epoxy was mixed first according to manufacturing mixing instructions. Tungsten powder was weighted for a mass equivalent to 10% volume ratio of the epoxy mix. Then, tungsten was added to the epoxy and mixed together. The structure was mechanically supported by a pin receptacle connector embedded in a custom-designed cuboid. The connector is not off the shelf but is very similar to a commercial pin receptacle connector (part number: 0461-3-15-21-27-04-0) by Mill-Max Manufacturing Corp., Oyster Bay, NY, United States of America. A batched fabrication protocol was employed such that an 8 by 8 pin receptacle board was diced into individual rectangular structures with the epoxy and PVDF layer on top of the pin receptacle connector. A 25 μm thick polyimide film was used as cover around the receiver structure for electrical insulation. A 10 μm thick copper foil (Goodfellow Cambridge Limited, Huntingdon, United Kingdom) was then wrapped around the insulating prism leaving the active face exposed. The face electrode of the PVDF film was connected to copper foil via silver epoxy, applied along the top surface edges. The fabrication protocol enables batch-wise production with a customized board of 64 pin receptacle connectors, improving fabrication efficiency and consistency of these receivers. 

**Figure 1:**
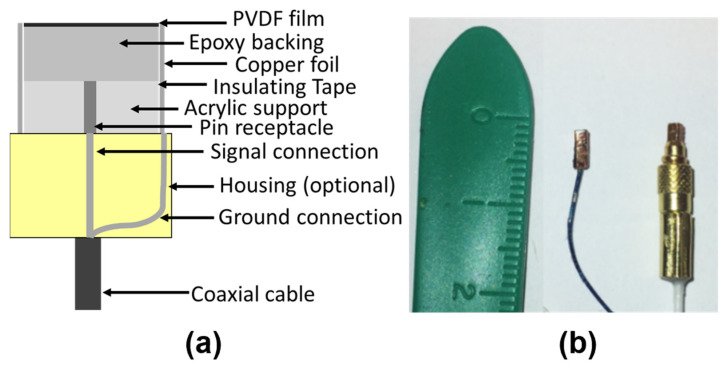
Schematic of the device (**a**) and fabricated receivers (**b**) with or without a standard MMCX connector as housing. A receiver without housing was connected to a micro coaxial cable.

An optional housing can be made using a standard MMCX connector with one end of male pin-plug type, as shown in Figure 1a. Alternatively, a micro coaxial cable can be used for electrical connections. Non-conductive epoxy (302-3M Epo-Tek^®^, Epoxy Technology, Billerica, MA, United States of America) was used to secure ground connections. Alternatively, the use of a male-to-male adapter (MMCX-JJ, Danyang Hengtong Electronics Co., Danyang, People’s Republic of China) enables rapid characterization by simply plugging the receiver into an MMCX female connecter. The finished element was then coated with approximately 10 μm of parylene C for electrical insulation and corrosion prevention. Figure 1b shows the receiver with and without optional housing. The material cost of the miniature receiver (including housing) is just under 4 USD.

### 2.2. Preamplifier Design

A small preamplifier with unity gain was constructed and enclosed within 3D printed acrylic housing to improve the signal-to-noise ratio (SNR). The operational amplifier selected (LMH6639, Texas Instruments, Dallas, TX, United States of America) has a −3 dB unity gain bandwidth of 190 MHz. The preamplifier was attached to a customized printed circuit board (PCB). A MMCX female jack connector (MMCX-C-XE, Danyang Hengtong Electronics Co., People’s Republic of China) was soldered onto the PCB for ease of individual receiver characterization. With the modularized design of the receiver assembly, the receiver and preamplifier can be fabricated and characterized separately in parallel for constructing an array. The circuit board geometry and housing can be customized according to the need and available space when designing an array.

### 2.3. Sensitivity Characterization

The receiver sensitivity was obtained by comparison with a standard National Physics Laboratory (NPL)-calibrated needle hydrophone. Briefly, the receiver (or hydrophone) was placed in an ultrasonic field and voltage output values were recorded at a given frequency. The same measurements were repeated for a calibrated hydrophone as the reference to calculate pressure. The receiver’s voltage-to-pressure sensitivity was then determined from the relationship between the recorded output voltage and pressure measured by the calibrated hydrophone [43]. 

**Figure 2:**
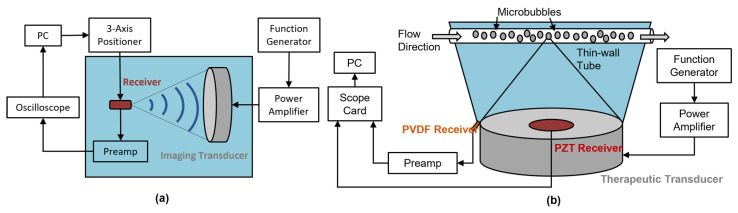
Sensitivity calibration setup (**a**) and bubble monitoring setup (**b**). In the calibration setup (**a**), a 3-axis positioner was controlled by a personal computer (PC) to adjust the receiver position until the location of maximum output signal was found, as shown on the oscilloscope. An imaging transducer was used to transmit sine waves sent out from the function generator and power amplifier, creating an acoustic field in the deionized and degassed water tank. In the benchtop bubble monitoring setup (**b**), the newly developed PVDF receiver with the in-house PZT receiver used for in vivo studies was compared by simultaneously capturing the acoustic emission from MBs. Preamp is a short form of preamplifier in both schematics.

The experimental setup is illustrated in Figure 2a. A three-axis positioner was used to adjust the position of the receiver inside a water tank in the field of the source transducer until the location of maximum output signal was found. The receiver sensitivity was measured across a wide range of frequencies (0.51–5.4 MHz) using two focused transducers (Olympus V389, nominal element size = 39 mm, f number = 1.5, focal length = 59 mm, center frequency = 500 kHz, and Olympus V392, nominal element diameter = 39 mm, f number = 2, focal distance = 78 mm, center frequency = 1 MHz) as source transducers in a tank of de-ionized and degassed water. The 500 kHz transducer was driven at 0.51, 1.7, 2.9, 4.1 MHz, and the 1 MHz transducer was driven at 1.1, 3.2, and 5.4 MHz. To ensure reproducibility and stability of the acoustic field, the source transducer was set to operate at its highest harmonic to define a smaller focus. In other words, the position of the receiver was moved to the highest harmonic focus in the alignment process prior to calibration, thus reducing spatial uncertainties between measurements.

A function generator (33500B series, Keysight, Santa Rosa, CA, United States of America) was used to send 50 sine wave bursts at each of the aforementioned frequencies with a burst period of 10 ms to a 53 dB radiofrequency power amplifier (NP-2519, NP Technologies Inc., Newbury Park, CA, United States of America). The output was used to drive the source transducer. Steady-state peak-to-peak received voltage values were acquired by taking the difference of maximum and minimum voltage output values for different driving voltages, using an oscilloscope (TDS3012, Tektronix, Beaverton, OR, United States of America). The oscilloscope was set to perform 16-waveform average for all measurements. The signals were then transferred to a computer for processing using MATLAB (Mathworks, Natick, MA, United States of America). 

The pressure at the focus was calculated by correlating voltage obtained from a needle hydrophone (HP series, diameter = 0.5 mm, Precision Acoustics, Dorchester, United Kingdom) to different input voltages and calibrated voltage-to-pressure sensitivities provided by NPL. Another commercial fibre-optic hydrophone (Precision Acoustics, UK) was also included for comparison. The calibration values of the fibre-optic hydrophone were provided with an estimated 13% standard error. The calibration values of the needle hydrophone were provided with standard errors measured at integer MHz frequencies. In the frequency range examined, the sensitivity of the needle hydrophone has an uncertainty of approximately 14.5%. The sensitivity was then determined from the ratio of output voltage to calculated pressure. The receiver sensitivity was corrected by preamplifier gain at each frequency. In short, the preamplifier gain was measured by taking the ratio of the output voltage and a known input voltage. A function generator was connected directly to the input of the preamplifier. The output of the preamplifier was connected to an oscilloscope. The output gain was then measured by taking the ratio of the output to the input voltage. Then, the receiver sensitivity was extracted by dividing the sensitivity values from preamplifier gain at each frequency. Spatial averaging artifacts were corrected by estimating the correction factor using the method described in [44]. The sensitivity of the fabricated receiver was compared with those of the needle hydrophone, and a previously described 5 mm diameter PVDF receiver [20] Moreover, a PZT receiver (diameter 16 mm, radius of curvature 60 mm, center frequency = 890 kHz, FUS Instruments Inc., Toronto, ON, Canada) used for most in vivo studies in our research lab was also characterized. Sensitivity was calibrated at 20 kHz intervals from 0.750 to 0.950 MHz, as well as at 1.16 MHz. Due to the differences in active area of the PZT and the PVDF receivers, sensitivity per unit area was calculated and compared.

### 2.4. Lower Dynamic Limit

It is important to quantify the minimum detectable pressure of the developed receiver for a comprehensive receiver characterization. The noise floor of a hydrophone assembly determines the minimum detectable pressure or the lower dynamic limit of the receiver, as defined by International Electrotechnical Commission Standard 62127—Part 3 Section A6.1. The minimum detectable pressure, or noise equivalent pressure, is determined by the noise level (in volts), detected by the hydrophone assembly over the bandwidth of interest. We compared the measured lower dynamic limit of the PVDF receiver with that of a fibre-optic hydrophone and a needle hydrophone at the same frequencies as Section 2.5 with Olympus V392 (1.1, 3.2, and 5.4 MHz).

### 2.5. Directivity Characterization

Receiver directivity, or acceptance angle, was measured using a similar experimental setup, as described in the sensitivity characterization section above. The only difference is that the receiver was mounted on a custom designed arm concentrically mounted to a fine-tuning rotation stage (Newport 481-A series, Irvine, CA, United States of America). Voltage measurements were taken at 5-degree intervals, for ±90 degrees from the center axis. Directivity patterns at three frequencies (1.1, 3.2, and 5.4 MHz) were examined.

For comparison, theoretical directivity patterns using the unbaffled model [45], assuming equivalent circular aperture [46] at different frequencies, were also calculated.

### 2.6. Benchtop Bubble Monitoring

A benchtop experiment was performed to examine the feasibility of acoustic detection using the PVDF receiver and an in-house PZT receiver with an adapted experimental setup similar to that previously described in [20]. A 0.05% *v*/*v* solution of Definity contrast agent (Lantheus Medical Imaging, North Billerica, MA, United States of America) was gravity fed through a thin-wall polytetrafluoroethylene tube (inner diameter = 0.8 mm, outer diameter = 1.4 mm, Goodfellow Cambridge Limited, United Kingdom) at a flow rate of approximately 1.7 mL/min. A spherically focused therapeutic transducer (center frequency = 0.58 MHz, third harmonic frequency = 1.78 MHz, diameter = 75 mm, radius of curvature = 60 mm) with a 20 mm center hole was used to sonicate the tube phantom, as shown in Figure 2b. The PZT receiver was coaxially mounted to the therapy transducer in the same configuration as in vivo studies conducted by our lab. The fabricated PVDF hydrophone was positioned at the same distance on the periphery of the therapeutic transducer, angled towards the geometric focus.

For each of the five sonications, 10 ms bursts of sine waves at 1.78 MHz were sent at a pulse repetition frequency of 1 Hz with increasing focal pressure (initial pressure = 0.100 MPa, each increment = 25 kPa). To examine if the PVDF receiver can identify the threshold pressure, which introduces nonlinear acoustic emissions from microbubbles, as determined by the PZT receiver, both receivers were set to simultaneously acquire 11 ms of data for each 10 ms burst, at a sampling rate of 20 MHz, captured using an oscilloscope card (Alazar Technologies Inc., Pointe-Claire, QC, Canada). Detailed data analysis was conducted using MATLAB.

The presence of characteristic frequencies of the subharmonic, second, and third harmonic of the transmit frequency (1.78 MHz) were evaluated by spectrum integration of a band ± 180 Hz at those frequencies. The threshold for subharmonic emission detection was defined as the sum of the arithmetic mean and three times the standard deviation of the subharmonic band at baseline. The baseline level was set by 10 consecutive acquisitions at initial pressure. The average pressure at which this threshold was reached for the PZT receiver was compared with the PVDF receiver.

## 3. Results

### 3.1. Comparison of PVDF Receiver and Commercial Hydrophones

**Figure 3:**
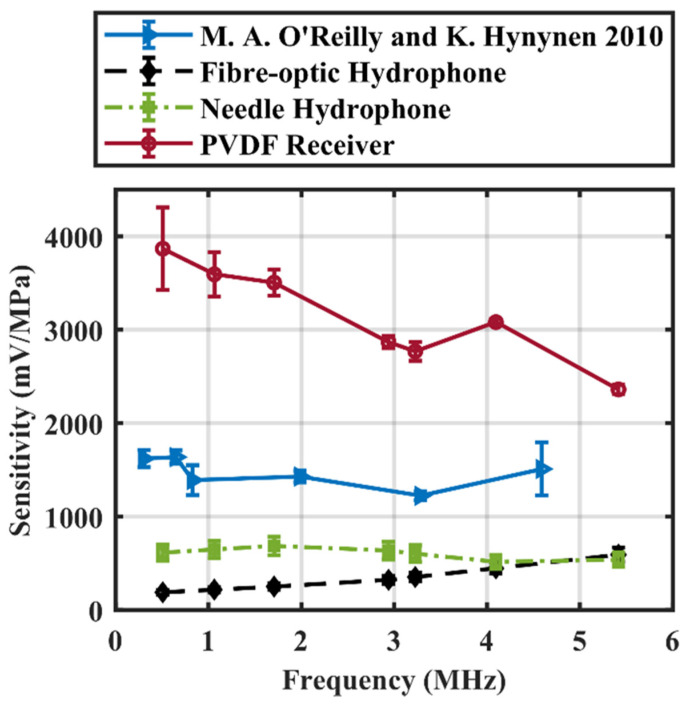
Sensitivity of a PVDF receiver from 0.51 to 5.4 MHz compared to that of a commercial fibre-optic hydrophone, the receiver developed by O’Reilly and Hynynen (Data from M.A. O’Reilly) [20], and a needle hydrophone. Preamplifier characteristics are corrected. Error bar indicates one standard deviation.

**Figure 4:**
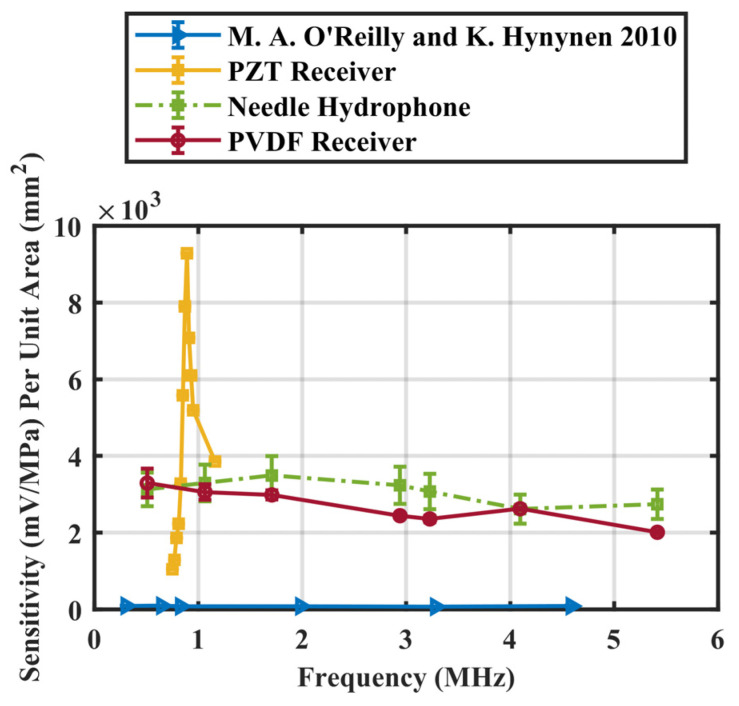
Sensitivity per unit area (mm^2^) of a PVDF receiver compared to the receiver by O’Reilly and Hynynen (Data from M.A. O’Reilly) [20], a needle hydrophone, and a PZT receiver fabricated in house (calibrated from 750 to 950 kHz and 1.16 MHz). Preamplifier characteristics are corrected. Error bar indicates one standard deviation.

The different receivers investigated in this paper are listed in Table 1. The receiver sensitivity with corrected preamplifier gain from 0.51 to 5.4 MHz is listed in Table 2 and compared with other receivers in Figure 3. The sensitivity of the receiver was up to 20 times that of the commercial fibre-optic hydrophone, 6.2 times that of the commercial needle hydrophone, and 2.4 times that of the previous developed PVDF receivers [20]. At 0.51 MHz, the PVDF receiver was 20 times that of the commercial fibre-optic hydrophone. The PVDF receiver had a greater variation in sensitivity in the lower frequency range due to the nature of the calibration method where a higher frequency focus of the transducer was used to guarantee spatial consistency. In general, the designed receiver is higher in sensitivity than the needle hydrophone in the sub-to-low MHz range and offers a fairly small element aperture for good spatial resolution if used as an element in an array. To normalize sensitivity across the different receivers examined, sensitivity (mV/MPa) per unit area (mm^2^) is demonstrated in Figure 4. The fabricated receiver outperformed the previously developed receiver [20] by more than an order of magnitude, across the entire frequency range. The PZT receiver had the highest sensitivity per unit area at 890 kHz. However, its narrow bandwidth limits its capability of detecting high frequency signals. The sensitivity per unit area quickly dropped to half of the maximum within 50 kHz of the center frequency. The normalized sensitivity of the PVDF receiver was also an order of magnitude higher than that of the PZT receiver, even at its natural resonance of 890 kHz.

**Figure 5:**
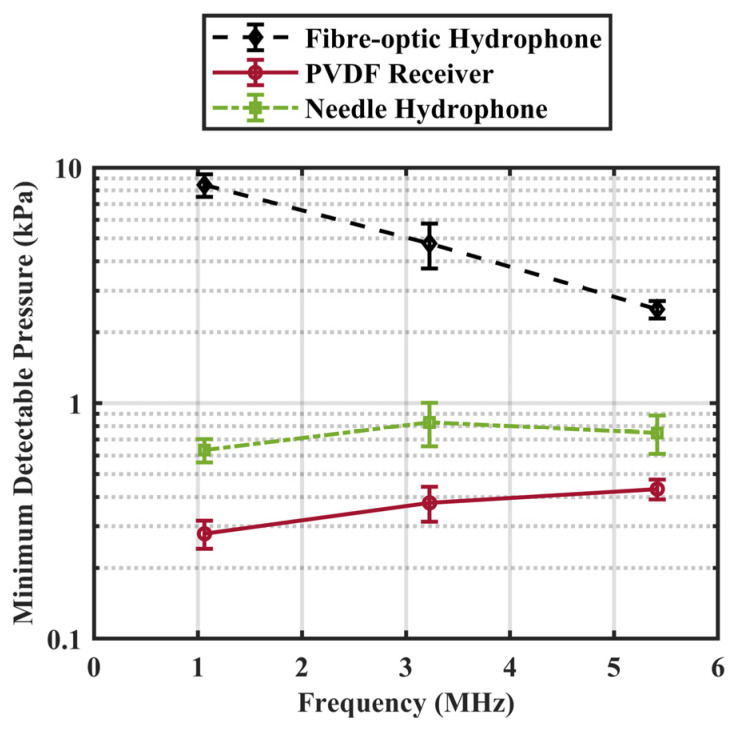
Minimum detectable pressure (kPa) as measured by a commercial fibre-optic hydrophone (Fibre optic 17–25, Precision Acoustics, United Kingdom), a needle hydrophone (HP series, Precision Acoustics, United Kingdom), and the PVDF receiver. Error bar indicates one standard deviation.

**Figure 6:**
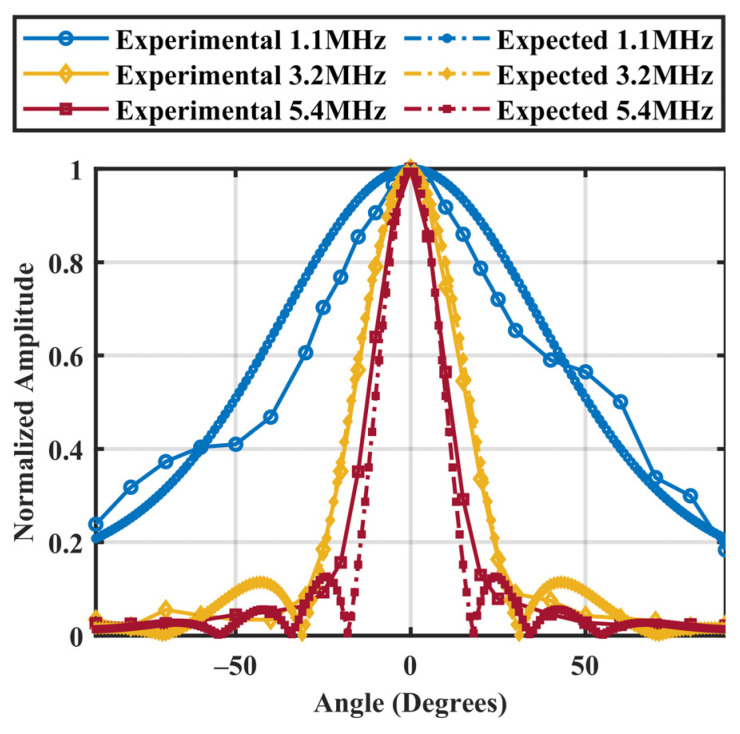
Theoretical and experimental directivity patterns of a PVDF receiver at discrete frequencies of 1.1, 3.2, and 5.4 MHz.

The lower dynamic limit of the receiver assembly (maximum of 0.39 ± 0.04 kPa at 5.4 MHz) was about an order of magnitude superior to that of the fibre-optic hydrophone, and approximately half of that of the needle hydrophone in the low MHz range (Figure 5). The developed PVDF receiver could detect pressure of 0.21 ± 0.03 kPa at 1.1 MHz, which can be greatly advantageous for detecting nonlinear sub- and/or ultraharmonic MB emissions at the low MHz, preclinically relevant frequencies. Comparing to symmetrical circular transducers, the beam pattern of a square transducer is given by a product of two sinc functions along the two axes of symmetry. To compare on an equivalent area basis, the directivity pattern of a receiver equivalent circular aperture is presented at each of the three frequencies. The directivity patterns are very similar to the theoretical predictions (Figure 6). At 1.1 MHz, the −6 dB acceptance angle is 54 degrees. With increasing frequency, the −6 dB acceptance angle drops to 13 degrees at 5.4 MHz. 

The batch-wise construction method enabled reproducibility and quality control over a large number of low-cost ultrasonic receivers. For a batch of 27 receivers, the receivers have an average 3.9 ± 1.1 V/MPa at 1.1 MHz and 1.53 ± 0.36 V/MPa at 5.4 MHz.

### 3.2. Validation on Benchtop Bubble Monitoring

**Figure 7:**
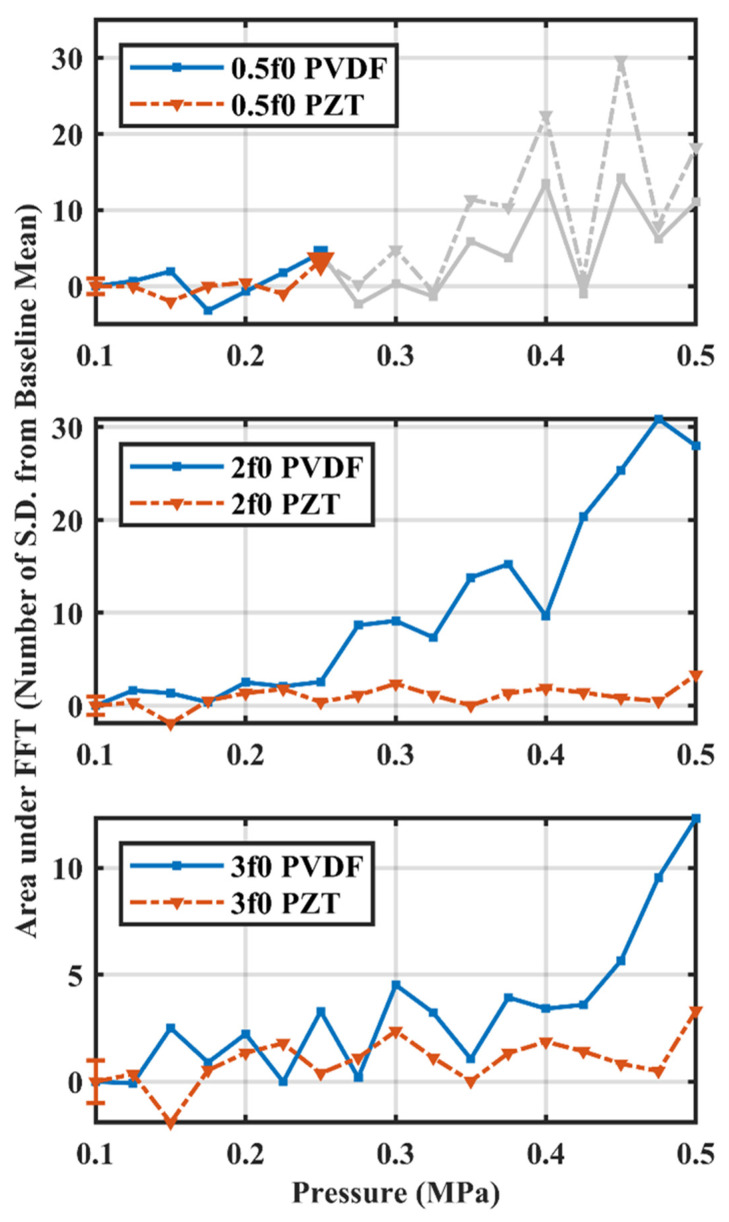
Microbubble emissions at ramping pressure levels captured by PZT and PVDF receivers up to 0.500 MPa. Area under the FFT was calculated as spectrum integration of ±180 Hz at the frequency of interest, normalized by standard deviation from the arithmetic mean of 10 baseline acquisitions. Pressure threshold would be 0.250 MPa for both PZT and PVDF receivers in this measurement. Grey lines indicate the signals acquired at pressures higher than the thresholding pressure. Error bars indicate one standard deviation of baseline acquisitions at 0.100 MPa.

The results from the benchtop bubble monitoring experiment demonstrate the fabricated PVDF receiver is capable of detecting nonlinear signals from MB emissions. Figure 7 shows the nonlinear signals acquired by both the PZT and PVDF receivers over increasing pressure, comparing subharmonic (0.5f0 = 890 kHz), second (2f0 = 3.56 MHz) and third (3f0 = 5.34 MHz) harmonic emissions normalized by baseline mean, respectively. As determined by the PZT-receiver-acquired signals, the pressure threshold would be 0.250 MPa. The same pressure threshold was suggested by the PVDF receiver.

**Figure 8:**
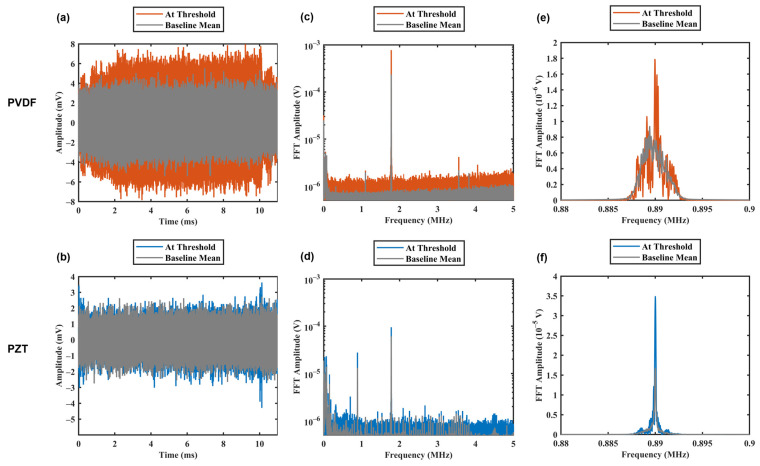
Cavitation signals acquired by PVDF (**a**,**c**,**e**) and PZT (**b**,**d**,**f**) in (**a**,**b**) time domain and (**c**,**d**) fast Fourier transform (FFT) amplitude at threshold pressures. Figures (**e**,**f**) show the change at the subharmonic, with seven times zero padding added to all data sets. Baseline mean is the arithmetic mean of 10 baseline acquisitions. Raw data were filtered with a fourth order Butterworth filter at the subharmonic frequency with 4000 Hz bandwidth.

Figure 8 shows the raw signals in time (a and b) and frequency (c and d) domains captured by a PVDF (a and c) and a PZT (b and d) receiver. Each raw data set was zero padded by seven times the data length and filtered at subharmonic frequency (0.5f0 = 890 kHz) with 4000 Hz bandwidth to clearly depict the FFT spectrum. Single-sided amplitude spectra of the PVDF (Figure 8e) and PZT (Figure 8f) at the threshold pressure are compared to the baseline mean. Both receivers demonstrated increases in signal strength at the subharmonic frequency comparing to the signal strength at baseline. Whereas PZT had a clear peak at the frequency of interest due to its narrow-banded characteristics, the broadband PVDF receiver was adequate in detecting characteristic emissions at the subharmonic frequency. Areas under the FFT spectrum (integrated ±180 Hz) at the threshold pressure were calculated to be 2.1 × 10^−5^ and 3.0 × 10^−4^ for PVDF and PZT, respectively. In both cases, the area under FFT values were higher than the sum of baseline means and three standard deviations, satisfying the thresholding conditions.

Greater second and third harmonic emissions were captured by the PVDF receiver than those of the PZT receiver. A more gradual increase in second harmonic emission strength was found in the signals captured by the PVDF receiver. At 0.5 MPa, the nonlinear second harmonic emission signal acquired by the PVDF receiver was more than two times the relative signal strength than that of the PZT receiver. The PVDF receiver was also capable of capturing emissions at the third harmonic. 

**Figure 9:**
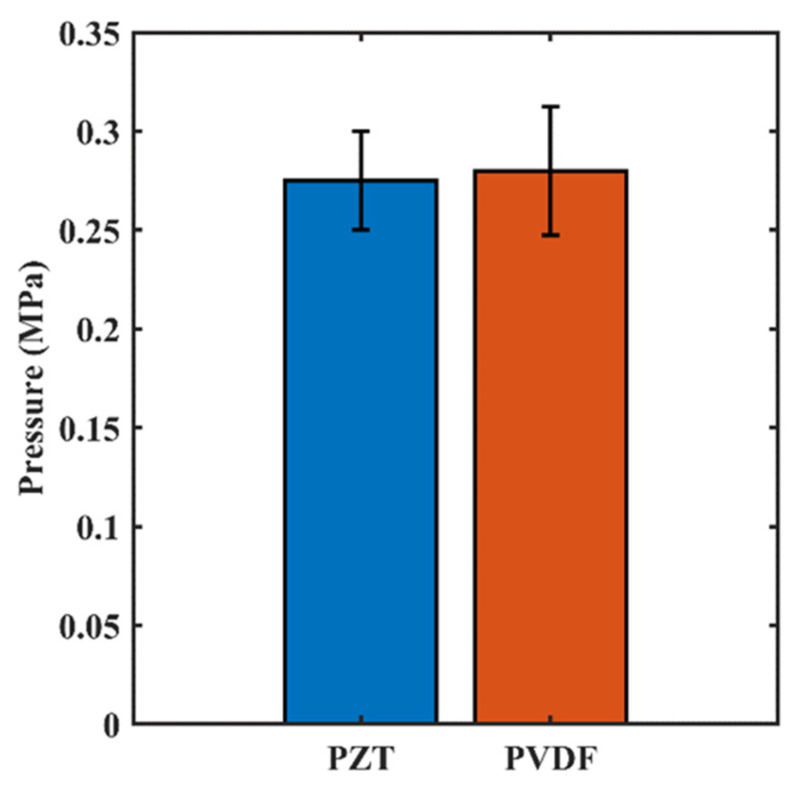
Pressure threshold levels of the PZT and PVDF receivers defined as sum of baseline mean and three standard deviations. Error bars indicate standard deviation of 5 tests. No statistical significance between the pressure values of PZT and PVDF was found (*p*-value = 0.7924).

The averaged pressure threshold, as determined by either receiver, is shown in Figure 9. Paired *t*-test found no statistical difference (*p*-value = 0.7924) between the averaged pressure threshold values of the two receivers.

## 4. Discussion

The fabrication protocol was optimized for mass-production. Receivers were built in a batch-wise fashion, allowing for reproducibility and quality control over 32 individual receivers (assuming a minimum of 50% yield). This fabrication protocol also lowers the cost of each receiver to under 4 USD, making it highly favorable for array construction. The variability in sensitivity is expected between receiver elements, just as it is commonly seen in other types of hydrophones. With more batches, a subset of receivers with less variability in sensitivity can be selected for array construction. 

In the context of passive cavitation detection, high sensitivity in the preclinically relevant low MHz range is essential for detecting sub- and/or ultraharmonic emissions. The enhanced sensitivity near 1 MHz, due to the use of the tungsten epoxy mix, is thus greatly favored. The gradual decrease in sensitivity at higher frequencies is likely due to the intrinsic resonant frequency of the 110 μm thick PVDF film at 10 MHz (λ/2 resonance). Using a thicker PVDF film as the active layer can be a way of increasing sensitivity by shifting resonance, though thicker PVDF films may not be readily available in the market. Nevertheless, care must be taken when choosing the appropriate film thickness as a significant drop in sensitivity is expected at frequencies higher than resonance.

The tungsten epoxy backing layer in this design may change the sensitivity profile in the low frequency range compared to water or air-backed designs [42]. It may also cause both intra- and inter-batch variability of the receivers to increase, due to inhomogeneity in hand-mixed epoxy in the fabrication process. In addition, an optimal tungsten volume ratio for achieving a specific backing layer impedance could be optimized in future work to tailor the receivers to applications interested in different frequency bands. Specific tungsten particle size may also be optimized for the frequencies of specific interest.

The receiver sensitivity was not examined at frequencies higher than 5.4 MHz. This is intentionally omitted as high frequency signals are greatly attenuated by skull bone, even in the case of thin mouse skull [47]. If a craniotomy was to be performed and high-frequency emissions can be obtained, the receiver sensitivity can be easily calibrated following the aforementioned method using imaging transducers at higher frequencies. However, element size must be taken into consideration at higher harmonics, or high-pressure applications [48], accounting for spatial averaging effects [43].

The well-known trade-off between receiver sensitivity and spatial resolution requires ultrasonic receivers to be designed according to their applications. Diminished sensitivity is expected when minimizing the sensitive area for receivers with higher spatial specificity. In the case of developing receivers for monitoring MB activities, the absolute sensitivity of receiver is as important as spatial resolution. Comparing to fibre-optic hydrophones, which are designed for high spatial resolution, the presented receiver is higher in sensitivity by an order of magnitude. On the other hand, the PVDF receiver is over 100 times smaller in the active area than the narrowband PZT receiver, thus providing much higher spatial resolution than if it was used in an array. In the case of a fibre-optic hydrophone, which has a higher sensitivity per unit area than any of the other receivers, it was not considered as a possible candidate in designing a receiver array due to the cost associated with scaling to an array of receivers. In other words, the PVDF receiver is a receiver capable of constructing an array specially designed for both sensitivity and spatial resolution in the use of monitoring ultrasound brain therapies.

Comparing to the hydrophone design by Jiang et al. [49], the receivers presented in this paper have comparable sensitivity in the range of −230 to −220 dB re 1 V/µPa. However, the length of the receiver presented here was reduced from 10 cm to 0.5 cm, to a maximum length of 2 cm with a MMCX connector. The compact design allows the realization of a potential hydrophone array with greater freedom in design geometry and maximizing the number of elements populating an array. Moreover, this receiver design has a smaller aperture (or 2.6 times smaller in active area), providing not only greater directional response but also higher spatial resolution if used in an array. As signal-to-noise ratio and spatial resolution increase with more receivers added to an array, the absolute sensitivity of each receiver becomes less of an issue. 

The upper limit of which this receiver can be used is not a concern for the application of acoustic monitoring of microbubbles in brains, where the pressure and frequency are limited to low MHz sonications at sub-MPa levels [50]. In the case of other applications, such as high intensity focused ultrasound, the upper limit of this receiver can be quantified, as specified in the International Electrotechnical Commission Standard 62127—Part 3, if needed. Other than the pressure threshold for physically damaging the receiver element, one should also consider linearity of the selected preamplifier for the validity of receiver output.

Differences between experimental and theoretical directivity patterns may be due to the fabrication process and the relative position of the receiver aperture to the rotational axis. Since silver epoxy was manually applied along the edge of the active surface to establish electrical connection, the aperture is not expected to be a perfect square. This irregularity in aperture can change the directivity pattern, depending on the silver epoxy geometry. Secondly, the receiver was only rotated along one axis. Although the receiver was positioned to be perpendicular to the imaging transducer with the best effort, slight angular misalignment may cause the misalignment along the rotational axis. This could cause distortions in measured directivity pattern at low frequencies in which the normalized amplitude is expected to gradually decrease as the receiver moves away from the beam axis. 

The estimated effective receiver element radius using the unbaffled model could also be a source of theoretical and experimental discrepancy at low MHz range. As frequency increases, the model better predicts the experimental directivity pattern as the error in the radius estimation declines. Although it was reported that the rigid piston model outperforms the unbaffled model [45], the unbaffled model is still a promising approach for which minimal computation effort is needed.

From the benchtop bubble experiments, the PVDF receiver demonstrates equivalent capability to detecting characteristic frequencies from MB emissions. The receiver can detect the same pressure threshold based on the onset of subharmonic emissions as that of the PZT receiver while being more sensitive to harmonic emissions. In other words, the PVDF receiver provides the whole acoustic spectrum of MB emissions and may provide new opportunities for control, such as peak broadening, which may be missed due to the narrow bandwidth of PZT receivers. This broadband characteristic enables more complex thresholding algorithms using MB emissions from multiple frequency bands and ultimately superior treatment control. In the context of array construction, signals from higher order harmonics may also be utilized for passive mapping purposes. A close-loop control algorithm can be constructed given the harmonic emissions after the pressure drop to achieve real-time acoustic monitoring. 

The large difference between sensitivity of the new receivers and receivers developed by O’Reilly and Hynynen in 2010 [20] is likely due to improved miniature design. A reduction in the active area by approximately 15 times greatly reduced receiver capacitance. Only with appropriately selected resistors in the preamplifier circuity will the response of the active layer be most effectively transferred. The sensitivity per unit area also shows that the new receiver demonstrated much higher normalized sensitivity than other receivers. Inexpensive miniaturized wideband receivers of higher sensitivity may have the potential for developing a preclinical receiver array for the passive acoustic mapping of MB activities [51].

To develop a receiver array, the directivity pattern of the current receiver provides sufficient estimation of the −6 dB acceptance angle for designing array geometry. The results indicate that a practical preclinical receiver array integrated in a hemispherical transmit array could allow bubble imaging at frequencies up to 5 MHz with an adequate imaging range to cover a whole mouse or rat brain. If the receiver is used in an array for super-resolution MB mapping, it is expected that single bubbles can be localized in capillaries due to the order of magnitude increase in the frequency compared with the previous work [52]. Moreover, a direct treatment control utilizing MB activity information can be developed as an alternative to MRI quantification methods [53]. This would allow microscopic control for the BBBM and thus provide a precise tool for brain research.

## 5. Conclusions

A miniature PVDF receiver for the acoustic monitoring of MB-mediated ultrasound brain therapy has been presented. Its sensitivity per unit area is comparable to the commercial needle hydrophone in the low MHz region; however, it has a design with great potential for constructing multi-element arrays. Its wide acceptance angle over the frequency range of interest suggests the receiver is suitable for use in a receiver array. With the low-cost, batch-wise fabrication protocol, the presented receiver has a strong potential for constructing a next generation pre-clinical receiver array enabling MB activity mapping. An array with MB mapping will allow precise localization and control of ultrasound brain therapy, enabling safe and accurate quantitative preclinical studies. The development of acoustic monitoring preclinical systems will increase the number of neurological indications for which FUS-induced BBBM is a viable and effective treatment option, ultimately allowing for more clinical trials and improving clinical outcomes.

## Figures and Tables

**Table 1:** Receivers of different designs.

	PZT Receiver	O’Reilly and Hynynen 2010	PVDF Receiver	Needle Hydrophone	Fibre-Optic Hydrophone
**Active Material**	PZT	PVDF	PVDF	PVDF	Fibre
**Active Area (mm^2^)**	201	17.8	1.2	0.2	79 μm^2^
**Frequency Range (MHz)**	0.7–1.1	0.3–4.6	0.5–5.4	0.1–20	0.25–50
**Sensitivity (Order of magnitude in mV/MPa)**	10^6^	10^3^	10^3^	10^2^	10^2^
**Connection**	Coax	Coax	Coax	Coax	Fibre-optic system
**Cost per receiver (USD)**	N/A	N/A	4	2087 *	389 **

* Quoted in 2019. ** Quoted in 2022; N/A—not reported.

**Table 2:** PVDF receiver sensitivity with corrected preamplifier gain.

Frequency (MHz)	Sensitivity ± 1 Standard Deviation (V/MPa)
0.51 *	3.87 ± 0.44
1.1 **	3.59 ± 0.24
1.7 *	3.51 ± 0.14
2.9 *	2.87 ± 0.07
3.2 **	2.77 ± 0.10
4.1 *	3.08 ± 0.04
5.4 **	2.36 ± 0.05

* Transducer Olympus V389 was used. ** Transducer Olympus V392 was used.

## Data Availability

Not applicable.
